# Highly Bendable and Durable Waterproof Paper for Ultra-High Electromagnetic Interference Shielding

**DOI:** 10.3390/polym11091486

**Published:** 2019-09-12

**Authors:** Fang Ren, Han Guo, Zheng-Zheng Guo, Yan-Ling Jin, Hong-Ji Duan, Peng-Gang Ren, Ding-Xiang Yan

**Affiliations:** 1 The Faculty of Printing and Packaging Engineering, Xi’an University of Technology, Xi’an 710048, China; 2 Shanxi Province Key Laboratory of Functional Nanocomposites, North University of China, Taiyuan 030051, China; 3 College of Polymer Science and Engineering, State Key Laboratory of Polymer Materials Engineering, Sichuan University, Chengdu 610065, China

**Keywords:** cellulose paper, silver nanowire, EMI shielding, waterproofness, flexibility

## Abstract

An efficient electromagnetic interference (EMI) shielding paper with excellent water repellency and mechanical flexibility has been developed, by assembling silver nanowires (AgNWs) and hydrophobic inorganic ceramic on the cellulose paper, via a facile dip-coating preparation. Scanning electron microscope (SEM) observations confirmed that AgNWs were interconnected and densely coated on both sides of the cellulose fiber, which endows the as-prepared paper with high conductivity (33.69 S/cm in-plane direction) at a low AgNW area density of 0.13 mg/cm^2^. Owing to multiple reflections and scattering between the two outer highly conductive surfaces, the obtained composite presented a high EMI shielding effectiveness (EMI SE) of up to 46 dB against the X band, and ultrahigh specific EMI SE of 271.2 dB mm^–1^. Moreover, the prepared hydrophobic AgNW/cellulose (H-AgNW/cellulose) composite paper could also maintain high EMI SE and extraordinary waterproofness (water contact angle > 140°) by suffering dozens of bending tests or one thousand peeling tests. Overall, such a multifunctional paper might have practical applications in packaging conductive components and can be used as EMI shielding elements in advanced application areas, even under harsh conditions.

## 1. Introduction

With the flourishing development of mobile communications and electronic devices, which has brought significant conveniences for people, the increasing electromagnetic (EM) waves that are inevitably emitted by these electronic devices can be harmful to human beings and cause the malfunction to other electronics close to them [1,2,3,4,5,6,7,8]. Thus, there is an urgent need to develop electromagnetic interference (EMI) shielding materials to reduce the EMI pollution. Metal and metallic alloy compounds are widely used as traditional EMI shielding materials due to their high conductivity. However, poor flexibility, high density, processing difficulties and susceptibility to corrosion, all limit its extensive application in electromagnetic shielding. Intrinsic conducting polymers, such as polyaniline, polypyrrole, and polythiophene and their derivatives (film or fabric) are considered to be suitable EMI shielding materials because of their good environmental stability. However, the difficulties in processing, the high cost and poor designability has hampered the large-scale application of these materials. Conductive polymer composites (CPC) with contained conductive nanofillers, such as carbon nanotubes (CNT), graphene, nanowires, nanorods, and transition metal carbide/carbonitride (MXene) have been viewed as advanced candidates for EMI shielding because of their being lightweight, easy to process, resistant to corrosion, and having tunable electrical conductivity [9,10,11,12]. Unfortunately, large shield thickness is typically required for commercially applicable CPC, thereby restricting the practical use of these materials in the aerospace sector, miniaturized portable devices and wearable sensors [13]. 

Recent studies have shown that metallizing the surface of insulating polymeric substrates is an important strategy for achieving ultrathin and ultra-efficient EMI SE performance [14,15]. To date, numerous surface metallization methods have been conducted to fabricate EMI shielding materials, including vapor deposition, electroless deposition [16,17], magnetron sputtering [18], and electroless plating [19,20]. Although satisfactory EMI shielding performance has been gained, weak interaction between metal and matrix often results in the detachment of metal particles, making the materials non-conductive in practical application [21]. Thus, preparing CPC with durable and superior EMI SE at small shield thickness remains an important mission. Fibrous materials with porous structure have been well demonstrated to enhance the interaction between the conductive fillers and the matrix [22]. In addition, the porous structure also improves the microwave absorption due to the multiple reflections of the microwaves [23]. 

Cellulose papers easily absorb water, due to the abundant hydroxyl groups in the cellulose chain [24], and their rough and porous structure creates a large interfacial area which allows for inorganic fillers dispersed in water to be easily deposited or coated onto the paper [24,25]. Besides, it is an excellent substrate for microfluidic transportation, without any additional pump, because of its own capillary forces [26]. Therefore, cellulose paper is a flexible substrate, which has potential for wearable electronic devices [27]. As conductive fillers, one-dimensional silver nanowires (AgNWs) are suitable and efficient for achieving high EMI performance, owing to their high aspect ratio and excellent electrical conductivity (6.3 × 10^7^ S/m), which is responsible for reflecting electromagnetic waves [3,28,29,30,31]. 

Herein, we have adopted a dip-coating process to fully realize the layered distribution of AgNWs on cellulose paper. The dip-coating technique has been considered to be very simple and efficient in manufacturing coatings, due to the short processing time, low cost and easy processability. The rough and porous nature of cellulose paper, as a flexible substrate, works well for AgNW deposition and adhesion. The dense AgNW layer is beneficial for high EMI shielding. In order to further enhance waterproofness and durability, hydrophobic inorganic ceramics were also used to form an enhanced coating on the surface of the AgNW layer, using the Mayer-rod coating method. The outer inorganic ceramic layer not only effectively prevents the AgNW from oxidizing and peeling off, but also prevents the external moisture from permeating. In this way, the resulting hydrophobic AgNW/cellulose (H-AgNW/cellulose) composite paper not only maintains the original nature of the cellulose paper with mechanical flexibility, but also possesses such significant properties as durable waterproofness and ultra-high EMI shielding. Such a multifunctional paper could have a wide application in packaging conductive components and can be used as EMI shielding elements in advanced application areas.

## 2. Materials and Methods

### 2.1. Materials

Cellulose filter paper (541, Whatman) with average pore size ~22 μm, 125 mm diameter, and ~154 μm thickness, acted as a neat cellulose substrate and was purchased from Sigma-Aldrich, India. The AgNW suspension (10 mg/ml) was purchased from Zhejiang Kechuang Advanced Materials Co., Ltd (Zhejiang, China), with an average diameter of 30 nm and an average length of 15 µm. Hydrophobic inorganic ceramic agent (GN-704, silicon dioxide as the main component, isopropyl alcohol as the solvent) was supplied by Guangzhou Yina Co. Ltd (Guangzhou, China).

### 2.2. Preparation of H-AgNW/Cellulose Composite Papers

**Figure 1:**
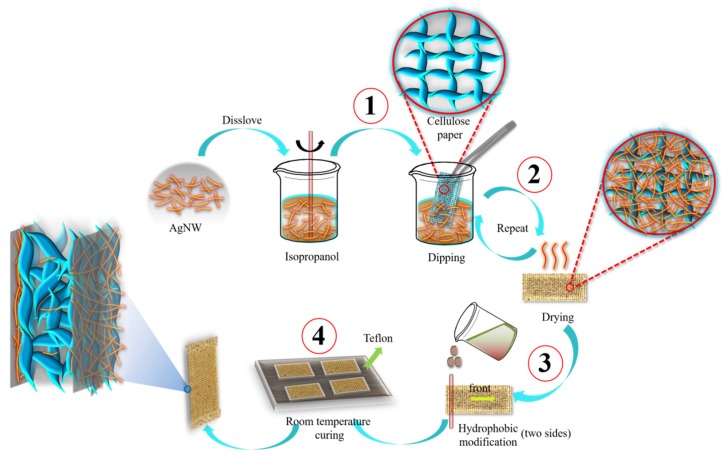
Fabrication process of H-AgNW/cellulose paper via a dip-coating process and hydrophobic modification.

The H-AgNW/cellulose composite papers were manufactured by dip-coating process, as shown schematically in Figure 1. First, in order to make the AgNW suspension more uniform, 5 mg/ml AgNW suspension was prepared by diluting a commercial AgNW suspension (10 mg/ml) with isopropyl alcohol. A neat pre-cut cellulose paper was dipped into the AgNW suspension for 3 s and then air-dried. This dip-coating process was repeated by 10 cycles to adjust the amount of AgNW coated on the cellulose paper. Then, the as-prepared AgNW/cellulose paper was fixed on a glass plate while the hydrophobic agent was dropped on the edge of the AgNW/cellulose paper, and a Mayer-rod #6 (RD Specialties, Inc., New York, USA) was rolled immediately over the drops to spread the hydrophobic agent over the AgNW/cellulose paper surface, followed by air-drying for 24 h. The same process was used to modify the other side. Finally, both sides of the H-AgNW/cellulose paper was obtained. The H-AgNW/cellulose papers were named as D-x, where x denotes the number of dip-coating cycles of AgNW.

### 2.3. Characterization

#### 2.3.1. Scanning Electron Microscopy

The morphology of the AgNW coated on the cellulose papers were characterized by observing the AgNW/cellulose composite paper surface and cross-section images with a field emission scanning electron microscope (Inspect-F, FEI, Hillsboro, USA), at an accelerating voltage of 5.0 kV. The microstructure morphology of the hydrophobic layer was observed on the H-AgNW/cellulose composite paper. Cross-sections for the scanning electron microscopy (SEM) observations were cut with a razor blade.

#### 2.3.2. Thermal Gravimetric Analysis 

Thermal gravimetric analysis (TGA) of the specimens was measured in a nitrogen atmosphere, using a Shimadzu TGA-50 thermogravimetric instrument. The temperature range employed was from 40 °C to 800 °C with a heating rate of 10 °C/min. The mass loading of AgNW in the as-prepared cellulose papers was calculated by TGA. In order to avoid the effect of hydrophobic inorganic ceramic agent, we measured the TGA of AgNW/cellulose composite paper to calculate mass loading of AgNW in the as-prepared composite paper.

#### 2.3.3. Contact Angle Measurement

The water contact angle (CA) of the H-AgNW/cellulose composite papers was measured using an optical contact angle (DSA30KRPUSS, Hamburg, Germany) to evaluate the hydrophobicity. Prior to measurement, the H-AgNW/cellulose papers were dried in vacuum in an oven at 50 °C for 6 h.

#### 2.3.4. Measurement of Electrical Conductivity

The electrical conductivities of the composite samples were measured using a Keithley electrometer, Model 4200-SCS (USA), according to a two-point method. Because hydrophobic inorganic ceramic is nonconducting, we measured the electrical conductivity of the AgNW/cellulose composite paper to characterize its electrical performance. Before the test, both ends of the rectangular specimens (22.86 mm × 10.16 mm) were coated with silver paste to reduce the contact resistance between specimens and electrodes. The electrical conductivity in the in-plane direction was calculated using the following equation:

(1) $$\sigma =\frac{1}{\rho }=\frac{1}{R}\times \frac{L}{A}$$

where σ is the electrical conductivity, ρ is the electrical resistivity, R is the resistance, A is the cross-sectional area, and L is the length or thickness of a sample between two electrodes. The thicknesses of AgNW/cellulose papers manufactured under different dip-coating cycles were measured using a digital thickness meter. Similarly, the electrical conductivity in the thickness direction was measured for the AgNW/cellulose papers. 

#### 2.3.5. EMI Shielding Characteristics

EMI shielding measurements were performed using the wave-guide method in the frequency range of 8.2–12.4 GHz by a network analyzer (Agilent technologies E8362B: 10 MHz-20 GHz). The intermediate frequency bandwidth was set at 1 kHz during the measurement and 201 points were collected for each specimen. Samples with 22.86 mm × 10.16 mm were placed in the specimen holder. The scattering parameters (S_11_ and S_21_) in the frequency range of 8.2–12.4 GHz were recorded to calculate the reflected power (R), transmitted power (T), absorbed power (A), EMI SE (SE_T_), microwave reflection (SE_R_) and microwave absorption (SE_A_), using the following equations:

(2) $$R=︱S_{11}{︱}^{2},T=︱S_{21}{︱}^{2}$$

(3) A=1−R−T

(4) $${\mathrm{SE}}_{R}\left(\mathrm{dB})=-10\;\log(1-R\right),\;{\mathrm{SE}}_{A}\left(\mathrm{dB})=-10\;\log(T/(1-R)\right)$$

(5) $${\mathrm{SE}}_{T}\left(\mathrm{dB}\right){=\mathrm{SE}}_{R}{+\mathrm{SE}}_{A}{+\mathrm{SE}}_{M}$$

where SE_M_ is the multiple microwave internal reflections, which can be negligible when SE_T_ is higher than 10 dB [32].

#### 2.3.6. Mechanical Properties Testing

The tensile properties of the composite samples were determined using an Instron universal tensile instrument (Model 5576, Instron Instrument, USA) according to ASTM standard D638: 1999 with a span length of 20 mm at a testing speed of 1.0 mm/min. The samples were tailored into rectangular strips, 100 × 10 mm^2^, and the thickness was measured separately for each sample. The average values of the tensile properties were presented with standard deviations for a minimum of five samples. The indentation stiffness (the bounce of bending 90°) was tested using an indentation stiffness tester (PN-CSI, China), with a 90° bending angle, and the distance from the fixture to the axis of rotation was 3 mm ± 0.1 mm. The samples were tailored into rectangular strips, 38 mm × 38 mm ± 0.1 mm, for a minimum of three samples. 

## 3. Results

### 3.1. The Morphology of H-AgNW/Cellulose Papers

**Figure 2:**
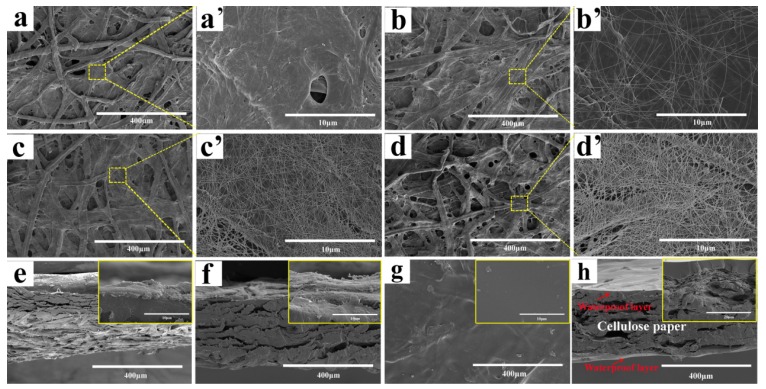
SEM images for surfaces of (**a**,**a’**) neat cellulose paper, (**b**,**b’**) D-1, (**c**,**c’**) D-5, (**d**,**d’**) D-10, and cross-section morphology of (**e**) neat cellulose paper, (**f**) D-5, respectively, SEM images of (**g**) surface and (**h**) cross-section morphology of AgNW/cellulose paper after coating with a hydrophobic layer.

The surface and cross-section SEM images of the pure cellulose paper, D-1, D-5 and D-10 are shown in Figure 2, respectively. A roughened surface and internal porous structure consisting of cellulose fibers was clearly observed, both from the surface and the cross-section of the pure cellulose paper (Figure 2a,a’,e). This is beneficial to the adsorption and fixation of AgNW on the surface and interior of the cellulose papers. The quite similar porous structure that also existed in the AgNW/cellulose papers indicates that the adsorption of AgNW did not affect the structure of the cellulose papers. However, individual AgNWs were clearly visible with a high aspect ratio, and were randomly interconnected on cellulose fibers on the high magnification SEM images of AgNW/cellulose papers. As the dip-coating cycles increased, the coated AgNW, without obvious aggregation on cellulose fibers, were densely connected to each other and formed a compact network among the AgNWs (Figure 2c’). As the dip-coating cycles increased to 10 (Figure 2d,d’), more AgNWs were joined to form denser isotropous AgNW networks, which provided crisscross transfer channels for the electron. The cross-section SEM images of D-5 (Figure 2f) show that AgNWs were strongly attached to cellulose fibers. This may be attributed to the roughened surface of the cellulose paper and the electrostatic interaction between the cellulose fibers and the AgNWs. The microstructure morphologies of the hydrophobic layer are shown in Figure 2g,h. As shown in Figure 2g, the cellulose fiber below the hydrophobic layer can be seen indistinctly, which indicates that the thickness of the hydrophobic layer is very small, ensuring the flexibility of the H-AgNW/cellulose paper. The magnification of the surface (Figure 2g inset) shows that it had good smoothness and a highly dense structure. Cross-sectional SEM images (Figure 2h) reveal the formation of a sandwich-like structure, with the encapsulation of AgNW/cellulose paper between the hydrophobic layers, and the Figure 2h inset shows that the thickness of the hydrophobic layer is about 6–8 μm. This typical sandwich-like structure gives the H-AgNW/cellulose paper unique characteristics, such as strong resistance to exfoliation, antioxidation and waterproofness.

### 3.2. The Electrical Property of H-AgNW/Cellulose Papers

**Figure 3:**
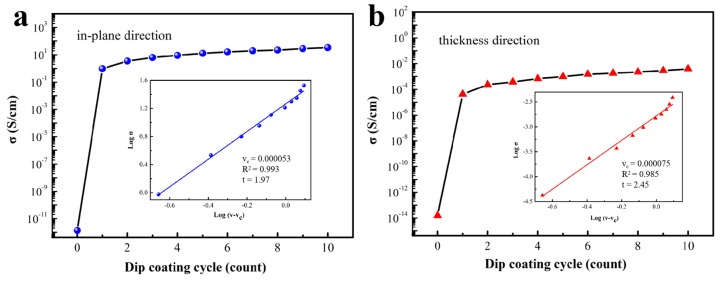
Conductivity of neat cellulose and AgNW/cellulose papers as a function of dip-coating cycles (**a**) in the in-plane direction and (**b**) the thickness direction.

The conductivities of H-AgNW/cellulose papers under different dip-coating cycles (1–10 cycles) are shown in Figure 3, and the corresponding data are summarized in Table 1 (the volume resistivity of AgNW/cellulose paper is shown in Table S1.). The mass loading of AgNW in the as-prepared cellulose papers was calculated by TG analysis (the corresponding results are shown in Figure S1). From Figure 3, it is evident that the electrical conductivity of the composite papers in both directions increases with increased dip-coating cycles and the conductivity of the in-plane direction is always much higher than in the thickness direction. For instance, the conductivity of the in-plane direction increased rapidly for the first dip-coating cycle from 10^–12^ of pure cellulose paper to 0.95 S/cm, which is more than four times that of the thickness direction. It means that the AgNWs are more densely coated on the cellulose paper surface, compared to the inner part of the paper. In addition, the improvement conductivity in the thickness direction also shows that some AgNWs infiltrated into the inside of the paper. The AgNW deposited on inner cellulose fibers through porous surface of paper decreases gradually in the thickness direction leading to a gradient structured composite, which is beneficial to the attenuation of the electromagnetic wave [33,34]. Thereafter, the conductivity of composite papers increased slowly, when the dip-coating cycle increased from 2 to 10. This suggests that the prepared composites exhibit a typical percolation behavior, which is usually evaluated with a power-law equation, i.e., $\sigma =\sigma_{0}{\left({v-v}_{c}\right)}^{t}$, where σ, σ_o_, v and v_c_ is the electrical conductivity of the composite, constant conductivity associated with the intrinsic characteristic of AgNW (σ_o_=6.3 × 10^5^ S/cm in this case), filler volume fraction, and the filler volume fraction at percolation threshold (the minimum contents of filler to construct the electrical path in composite), respectively. *t* represents a critical exponent related to dimensionality. The fitted curve of Logσ vs. Log(v-v_c_) is listed in the inset of Figure 3a and b. The electrical percolation in-plane and in thickness direction are as low as 0.0053 vol% and 0.0075 vol%, respectively, which is consistent with the result of Martin et al. (0.0021 wt%) [35] and superior to other references (Table S2). This indicates the conductive networks are effectively established in the prepared composites. Once the conductive network is formed, the conductivity is indistinctive with the filler increasing [36]. In addition, the values of *t* in the in-plane direction is 1.97, which is considered to be the 2D electrical percolation, while in the thickness direction, *t* is 2.45, showing a 3D characteristic, due to a small amount of AgNW penetrating into the paper [37]. On the whole, the conductivity of prepared AgNW/cellulose paper with 10 dip-coating cycles reaches 33.69 S/cm in the in-plane direction and 3.87 × 10^–3^ S/cm in the thickness direction, which completely meets the practical application for EMI shielding [12,38]. 

### 3.3. EMI Shielding Property of H-AgNW/Cellulose Papers

**Figure 4:**
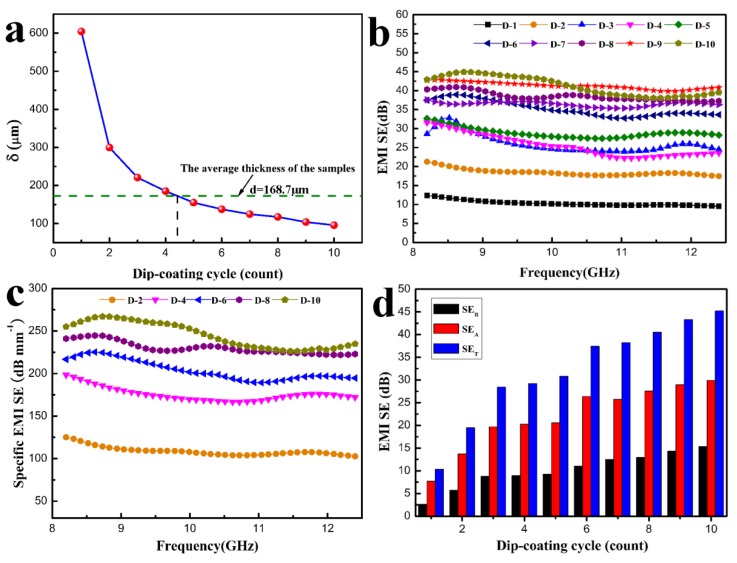
(**a**) Skin depth of the H-AgNW/cellulose papers as a function of dip-coating cycles at the frequency of 8.2 GHz. (**b**) electromagnetic interference shielding effectiveness (EMI SE) properties of H-AgNW/cellulose papers. (**c**) Specific EMI SE of typical composites. (**d**) Comparison of SE_T_, SE_A_, and SE_R_ at the frequency of 9.0 GHz for the composites with various dip-coating cycles.

It is well known that EMI shielding performance is closely related to electrical conductivity. The higher electrical conductivity of the composite paper is expected to be beneficial to EMI shielding performance [1,6]. In addition, skin depth (δ) of a material is another important physical parameter for the shielding ability. The δ value of the H-AgNW/cellulose papers with various dip-coating cycles can be calculated by the equation:

(6) $$\delta =\sqrt{\frac{1}{\pi f\mu \sigma }}$$

when $\sigma \gg 2\pi f\varepsilon_{0}$, $\mu =\mu_{0}\mu_{r}$, where ε_0_ is the permittivity of free space (equal to 8.854 × 10^–12^ F/m), *f* is the frequency of the EM wave, σ is the conductivity (in-plane direction in this work) of the samples, *μ, μ_r_* and *μ_0_* are the magnetic permeability, relative magnetic permeability (*μ_r_* = 1 in this case), and the permeability of free space (equal to 4*π* × 10^–7^ H/m), respectively [39]. Figure 4a shows the δ value at 8.2 GHz for the H-AgNW/cellulose papers. The δ value of D-1, D-5 and D-10 are 604.4, 155.1 and 95.8 μm, respectively. It is found that the δ value of the papers (after more than five dip-coating cycles) is much thinner than the thickness of our average testing sample of 168.7 μm. The high-performance EMI shielding was usually evident for samples when the thickness was beyond the δ [40,41].

**Figure 5:**
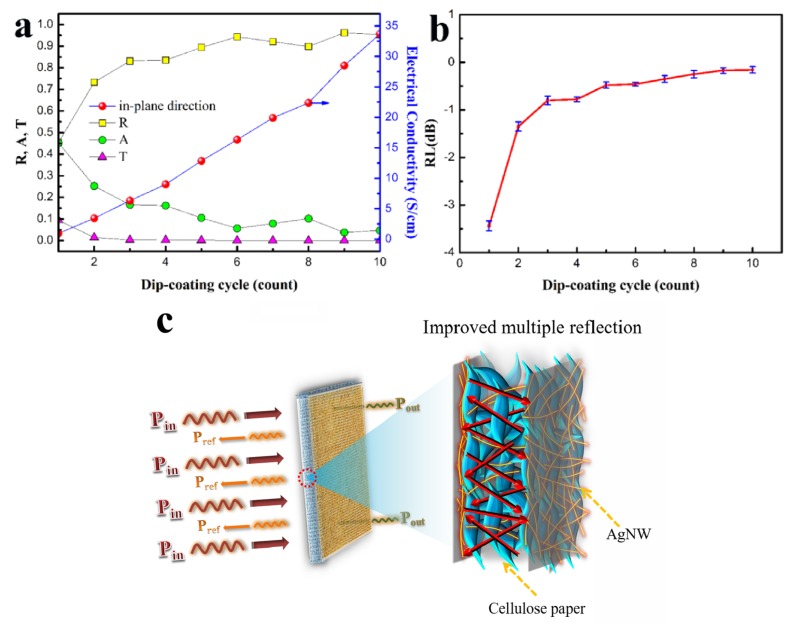
(**a**) Coefficients of reflection, absorption, and transmittance at 10.0 GHz and their dependence on electrical conductivity. (**b**) Reflection loss of the H-AgNW/cellulose composite papers as a function of dip-coating cycles at 10.0 GHz. (**c**) Schematic of electromagnetic microwave dissipation in H-AgNW/cellulose composite papers.

Figure 4b shows the EMI SE properties of H-AgNW/cellulose papers under different dip-coating cycles (1–10 cycles) in X-band (EMI SE values of neat cellulose paper are shown in Figure S2). Obviously, the H-AgNW/cellulose papers show a strong EMI shielding ability, which increases with the number of dip-coating cycles. As expected, the maximum EMI SE of composite paper reaches up to 10 dB with one dip-coating cycle, and the composite paper displays a satisfactory EMI SE 21.5 dB with only two dip-coating cycles, which meets the target requirement for commercial application [42]. This indicates that the dip-coating process is very efficient in forming a conductive network of AgNW on cellulose fibers. The EMI SE increases abruptly after more than five dip-coating cycles, which is consistent with the results of δ. Upon increasing the dip-coating cycle to 10, the H-AgNW/cellulose composite paper demonstrates an impressive EMI SE of 46.1 dB. By taking the thickness into consideration, a strikingly high specific EMI SE (EMI SE/thickness) of 271.2 dB mm^–1^ can be achieved for the D-10, with a low AgNW volume fraction of only 1.24 vol% (Figure 4c), which is superior to reference materials in terms of electrical conductivity and thickness (Table 2). The electromagnetic shielding properties of samples before and after hydrophobic modification were also investigated, as shown in Figure S3. Negligible variation on the EMI SE implies that the ceramic coating has no effect on the EMI SE of the samples. We speculate that the high SE_T_ values of the H-AgNW/cellulose composite papers can be attributed to the high intrinsic electrical conductivity of AgNW, as well as two highly conductive AgNW surface layers and gradient structure, formed in the cellulose papers by the dip-coating process. In order to evaluate the dominant shielding mechanism of H-AgNW/cellulose paper, the SE_T_, SE_A_ and SE_R_ of composite paper with different dip-coating cycles, at the frequency of 9.0 GHz, were calculated and results are listed in Figure 4d. The SE_T_ and the SE_A_ are obviously enhanced with increasing dip-coating cycles. It is worth mentioning that the SE_A_ is higher than that of SE_R_. Under 10 dip-coating cycles, the value of SE_A_ reaches 29.9 dB, almost 70.3% of SE_T_, indicating the absorption dominant shielding mechanism, rather than reflection, in the prepared composites. However, according to the power balance of transmission (T), reflection (R), and absorption (A) versus incident power, which is shown in Figure 5a, the actual amount of power shielded by reflection is larger than that due to absorption. For example, for the H-AgNW/cellulose paper with 10 dip-coating cycles, the R, A and T are respectively 0.953, 0.047 and 8.8 × 10^–5^, which means that 95.3% of the total EM wave power is reflected, and the value of SE_A_ reaches 29.9 dB, indicating that 99.9% of the EM wave power into the inner part of the composite (4.7% of the total EM wave power) is absorbed.

In order to clarify the major EMI shielding mechanisms of the AgNW/cellulose paper, the reflection loss (RL) is calculated based on the S-parameters obtained from the vector network analyzer according to equation:

(7) $$\mathrm{RL}\left(\mathrm{dB}\right)=10\log{\left|S_{11}\right|}^{2}$$

Figure 5b gives the plot of reflection loss versus dip-coating cycles. As shown in Figure 5b, the RL of the composite papers increases with the increasing dip-coating numbers. The RL almost reaches the standard line (0 dB) at 10 dip-coating cycles. In this respect, the high shielding performance of the AgNW/cellulose paper should be attributed to the reflection phenomena, which increases with the increasing composite conductivity. Thus, the contribution of reflection to the overall EMI SE is much larger than that of the absorption when the composite is highly conductive.

To comprehend the EMI shielding mechanisms of H-AgNW/cellulose paper more intuitively, a schematic of EM dissipation in H-AgNW/cellulose paper is shown in Figure 5c. The good electromagnetic shielding properties of the H-AgNW/cellulose paper may be explained by the following reasons. On the one hand, the two highly conductive AgNW surface layers formed in cellulose papers by the dip-coating process cause large surface reflection. On the other hand, the cellulose layer, as a spacer between the two outer highly conductive surfaces, with a small amount of infiltrated AgNW, can consume the incident waves through multiple reflections and scattering inside the composite paper [54,55,56].

### 3.4. Waterproofness and Durability

**Figure 6:**
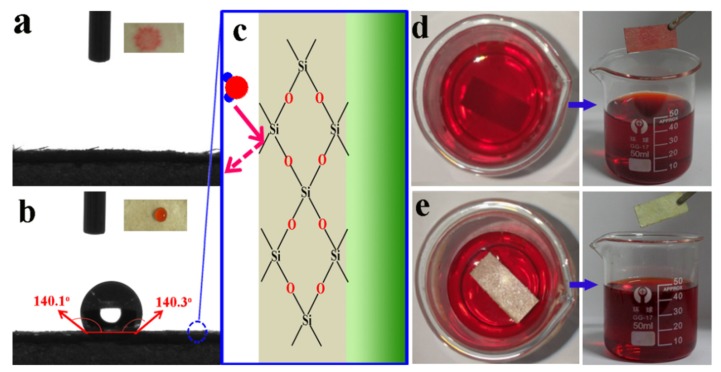
Wettability of the AgNW/cellulose and H-AgNW/cellulose paper. (**a**) Water drop on the AgNW/cellulose paper, (**b**) water drop on the H-AgNW/cellulose paper, (**c**) the waterproof mechanism of H-AgNW/cellulose paper (**d**) AgNW/cellulose paper is polluted when immersed in dirty water (congo red dissolved in water), (**e**) the H-AgNW/cellulose paper is antifouling to dirty water.

**Figure 7:**
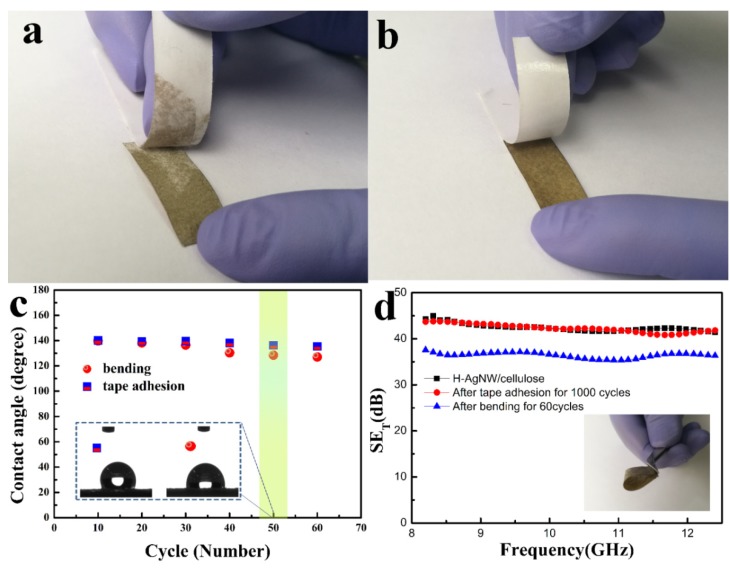
Tape adhesion test on the AgNW/cellulose paper before and after coating with a hydrophobic layer. (**a**) AgNW/cellulose paper, (**b**) H-AgNW/cellulose paper after the peeling off test for 60 cycles. (**c**) Change in contact angle (CA) of water droplet during multiple abrasion and bending cycles (inset photograph shows static CA of water on mechanically tested surfaces), and (**d**) EMI SE variation of H-AgNW/cellulose paper before and after multiple abrasion and bending cycles. The inset shows the bending state.

Compared to the plastics, the inherent highly hydrophilic character of cellulose fibers exhibit poor waterproofness, which would decrease the adhesion of AgNW on cellulose paper, and destroy the conductive network. Thus, waterproofness is a key prerequisite in the process of usage to enable the composite papers to work steadily [57,58]. In this work, hydrophobic inorganic ceramic was used to form an enhanced coating on the surface of the AgNW layer. The obtained H-AgNW/cellulose paper exhibits excellent hydrophobicity. As shown in Figure 6a, the water droplet penetrates inside of the paper immediately with a water CA approaching 0°. By contrast, the water CA dramatically increases to 141° after the coating with a hydrophobic layer (Figure 6b), indicating an extraordinary waterproofness. As Figure 6c shows, hydrophobic agent is cured into the SiO_x_ hydrophobic network, which effectively blocks the entry of water molecules. In addition, the H-AgNW/cellulose paper also exhibits an antifouling function in dirty water. As shown in Figure 6d, the original AgNW/cellulose paper is dyed red by immersing it into dirty water within a short time (about 3 seconds), whereas the H-AgNW/cellulose paper remains unchanged, showing a remarkable antifouling ability in dirty water (Figure 6e). Furthermore, the H-AgNW/cellulose paper also maintains noteworthy mechanical flexibility and resistance in comparison to conventional mechanics. Figure 7 shows digital comparison pictures of the peeling tests of both AgNW/cellulose paper and H-AgNW/cellulose paper by adhesive tape. The deposited AgNW was easily peeled off from the cellulose paper substrate (Figure 7a) by adhesive tape. By comparison, the H-AgNW/cellulose paper maintained superior adhesion, even after the peeling off test was conducted for 1000 cycles (see Figure 7b). It can be noted that the intrinsically fragile AgNW network was anchored into the hydrophobic inorganic ceramic layer, which effectively prevents AgNW stripping from cellulose paper substrate. Because of the thin hydrophobic coating by the Mayer-rod coating method, the H-AgNW/cellulose papers are highly flexible that can be bent to a large degree without breaking. For the above-mentioned tests, the CA of water droplets was measured after each of the 10 consecutive cycles and plotted in Figure 7c. Negligible variation in static CA over the mechanically perturbed surfaces implies the stability of the coating. The EMI shielding reliability to resist external forces is also examined in the EMI shielding robustness of the H-AgNW/cellulose papers. The EMI SE of the H-AgNW/cellulose paper remains essentially unchanged after tape adhesion for 1000 cycles (Figure 7d). The H-AgNW/cellulose paper maintains 85% EMI SE even after 60 bending cycles, which demonstrates the excellent EMI shielding reliability. Similarly, in order to further comprehensively evaluate the EMI shielding durability of the H-AgNW/cellulose papers, EMI SE variation of the papers undergoing other ultrasonic and environmental tests were also investigated, and the corresponding results also displayed in Figure S4. No obvious EMI SE degradation was observed for Dip-9 and the water in the bottle remained clear and transparent after ultrasonic treatment for 60 min (Figure S4a), indicating the mechanical robustness and fastness of the hydrophobic coating. Moreover, negligible variation in static CA over the mechanically perturbed surfaces implies the durability of the coating with environmental effects (Figure S4b). The developed H-AgNW/cellulose papers exhibit a strong EMI shielding, high waterproofness, and EMI shielding reliability, which provides significant potential as a high-performance waterproof EMI shielding material.

### 3.5. Mechanical Properties of Pure AgNW/Cellulose Paper and the H-AgNW/Cellulose Paper

**Figure 8:**
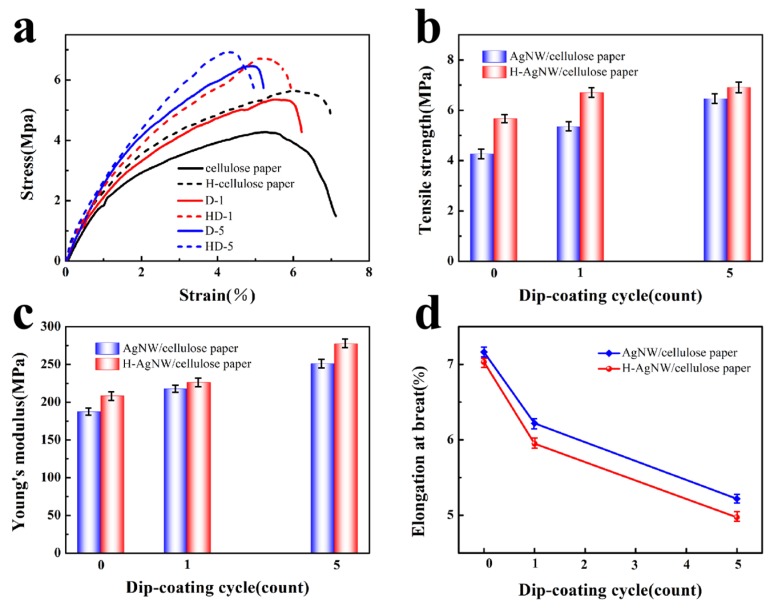
(**a**) Typical stress–strain curves, (**b**) Tensile strength, (**c**) Young’s modulus, (**d**) Elongation at break of AgNW/cellulose paper and the H-AgNW/cellulose paper as a function of dip-coating cycles.

Good mechanical properties are also essential for flexible EMI shielding materials. The tensile test of the composite papers was executed and the results are shown in Figure 8. The tensile strength and Young’s modulus of samples are both enhanced with the increase of dip-coating cycles and the surface hydrophobic modification. With the five dip-coating cycles (D-5), the tensile strength of pure cellulose paper increased from 4.27 to 6.46 MPa and the Young’s modulus increased from 187.3 to 251.2 MPa (Figure 8b,c). Such obvious strengthening and toughening effects may be attributed to the excellent strength of AgNW, as well as good electrostatic interaction between the AgNW and the cellulose paper. However, the elongation at break decreased from 7.16% to 5.21% due to the deformation restriction of the cellulose fiber by AgNWs. In addition, the mechanical properties of the paper are also effectively improved with the addition of the hydrophobic layer. For example, the tensile strength and Youngs modulus of the hydrophobic AgNW/cellulose paper with 5 dip-coating cycles increased to 6.91 MPa and 277.5 MPa, respectively. The remarkable increase of tensile strength and Young’s modulus for H-AgNW/cellulose paper resulted from the high strength of the inorganic hydrophobic layer, which also caused a slightly decrease in elongation at break. 

We also investigated the indentation stiffness of the samples and the result is shown in Figure S5. With the dip coating cycles of AgNW increasing, indentation stiffness of the samples increased slightly, while a large increment was obtained for H-AgNW/cellulose papers. This may be ascribed to the excellent hardness of AgNW and inorganic hydrophobic layer as well as increment in thickness of the composite papers. Regardless, the prepared H-AgNW/cellulose paper exhibits not only good electromagnetic shielding effectiveness, but also excellent mechanical properties. Together with the high waterproofness and EMI shielding reliability, this approach provides a novel idea for manufacturing new high EMI shielding materials with both toughness and waterproofness.

## 4. Conclusions

Multifunctional flexible waterproof papers with efficient EMI shielding performance were easily fabricated via an efficient and simple dip-coating process. The continuous interconnecting networks of the AgNW through the cellulose paper provide good conductivity and high EMI SE under low AgNW content. The composite paper, with a thickness of only 0.17 mm, can achieve an excellent EMI SE of up to 46 dB against the X band, and ultrahigh specific EMI SE of 271.2 dB mm^–1^. Moreover, the prepared H-AgNW/cellulose composite paper could also maintain high EMI SE and extraordinary waterproofness (water contact angle > 140°) by suffering dozens of bending tests or one thousand peeling tests. The obtained composite paper also exhibits excellent mechanical properties. This work provides a novel and facile methodology to fabricate multifunctional paper with lightweight, flexible and waterproofness for highly efficient EMI shielding applications.

## Figures and Tables

**Table 1:** Structural Parameters and Electrical Properties of Neat Cellulose and AgNW/Cellulose Papers Manufactured by a Dip-Coating Process.

Sample Code	Average Thickness (μm)	AgNW Content (wt %)	AgNW Content (vol %)	Sample Weight (mg)	AgNW Area Density (mg/cm^2^)	Conductivity (Inplane Direction) (S/cm)	Conductivity (Thickness Direction) (S/cm)
Neat paper	154.6 ± 3.9	0	0	26.0 ± 0.05	0	1.34 × 10^–12^	1.54 × 10^–14^
D-1	157.3 ± 2.9	1.52	0.22	26.4 ± 0.04	8.62 × 10^–2^	0.95	4.23 × 10^–5^
D-2	159.7 ± 3.5	2.80	0.41	26.8 ± 0.04	1.61 × 10^–1^	3.44	2.31 × 10^–4^
D-3	160.8 ± 3.2	4.00	0.59	27.1 ± 0.03	2.33 × 10^–1^	6.33	3.71 × 10^–4^
D-4	162.5 ± 3.3	4.86	0.73	27.4 ± 0.03	2.86 × 10^–1^	9.01	6.71 × 10^–4^
D-5	164.9 ± 3.4	5.57	0.84	27.6 ± 0.03	3.31 × 10^–1^	12.84	9.69 × 10^–4^
D-6	166.4 ± 3.1	6.56	0.99	27.8 ± 0.04	3.93 × 10^–1^	16.32	1.49 × 10^–3^
D-7	168.2 ± 3.2	7.05	1.07	27.9 ± 0.05	4.25 × 10^–1^	19.90	1.79 × 10^–3^
D-8	169.1 ± 3.4	7.48	1.14	28.1 ± 0.03	4.53 × 10^–1^	22.36	2.22 × 10^–3^
D-9	169.6 ± 3.5	7.76	1.19	28.2 ± 0.03	4.71 × 10^–1^	28.49	2.85 × 10^–3^
D-10	170.1 ± 3.7	8.06	1.24	28.2 ± 0.04	4.91 × 10^–1^	33.69	3.87 × 10^–3^

**Table 2:** Comparison of the EMI shielding performance of various Ag filled or coated materials.

Material	Filler Content	Thickness (mm)	Conductivity (S/cm)	EMI SE (dB)	Specific EMI SE (dB/mm)	Ref.
Ag/Poly(lactic acid)	5.89 vol%	1.5	2.54	50 (8.2–12.4 GHz)	33.3	[43]
AgNW/MWCNTs/cellulose	2 wt%	0.16	2.83	23.8 (1GHz)	176.9	[44]
Porous AgNW/Polyurethane	28.6 wt%	2.3	-	64 (8.2–12.4 GHz)	27.8	[45]
Ag@FRGO/Polyurethane	5 wt%	2	25.52	35 (8.2–12.4 GHz)	17.5	[46]
CA/AgNW/PU	-	~0.33	8.4	31.3 (8.2–12.4 GHz)	94.8	[47]
Ag/MWCNT/Melamine foam	3.7 wt%	2	2.53	68.1 (0.05–18 GHz)	34.0	[48]
AgNW /Polyimide foam	8.7 wt%	5.0	-	21 (200 MHz)	4.2	[49]
MWNTs/rGO@Ag/PCL	3 wt% + 5 wt%	5.5	~10^–2^	37 (18 GHz)	6.7	[50]
(GO/PPy)/fabric	-	2.236	-	22.2 (8.0–12.0 GHz)	9.93	[51]
Double coated PPy/Ag/PET fabric	-	0.45	-	13.5 (15–3000 MHz)	30	[52]
CEF-NF nonwoven fabric	40 wt%	0.175	15.969	30.29 (30–1500 MHz)	173.1	[53]
H-AgNW/cellulose	1.21 vol%	0.17	33.69	46.1 (8.2–12.4 GHz)	271.2	This work

## Supplementary Materials

The following are available online at https://www.mdpi.com/2073-4360/11/9/1486/s1, Figure S1: TG analysis of the as-prepared cellulose papers under different dip-coating cycles (0–10 cycles), measured from 30 °C to 800 °C. Figure S2: The EMI SE properties of neat cellulose papers. Figure S3: The EMI SE values of neat cellulose paper (a) and the sample D-1 (b) before and after hydrophobic modification. Figure S4: (a) EMI SE in the range of 8.2–12.4 GHz (X band) of the H-AgNW/cellulose after washing three days; (b) Change in CA of water droplet during exposure to air for 5 days (Inset photograph showing static CA of water on mechanically tested surfaces). Figure S5: The stiffness test of the cellulose paper and the H-AgNW/cellulose paper. Table S1: Volume resistivity of Neat Cellulose and AgNW/Cellulose Papers Manufactured by a Dip-Coating Process. Table S2: Comparison of the lowest electrical percolation thresholds in the literature.
