# Supplementary material for: Highly Bendable and Durable Waterproof Paper for Ultra-High Electromagnetic Interference Shielding
Source: Polymers (Basel). 2019 Sep 12;11(9):1486. doi: 10.3390/polym11091486 (PMC6780577; doi:10.3390/polym11091486)
Supplement: Supplementary file 1 [file polymers-11-01486-s001.pdf]

Supplementary Materials: Highly Bendable and Durable Waterproof Paper for Ultra-high Electromagnetic Interference Shielding

Fang Ren, Han Guo, Zhengzheng Guo, Yanling Jin, Hongji Duan, Penggang Ren, Ding-Xiang Yan

Table S1. Volume resistivity of Neat Cellulose and AgNW/Cellulose Papers Manufactured by a Dip-Coating Process

Sample code	volume resistivity (inplane direction) (Ω·m)	volume resistivity (thickness direction) (Ω·m)
Neat paper	7.46×10^{9}	6.49×10^{11}
D-1	0.01	236.4
D-2	2.91×10^{-3}	43.29
D-3	1.58×10^{-3}	26.95
D-4	1.11×10^{-3}	14.90
D-5	7.79×10^{-4}	10.32
D-6	6.13×10^{-4}	6.71
D-7	5.02×10^{-4}	5.59
D-8	4.47×10^{-4}	4.50
D-9	3.51×10^{-4}	3.51
D-10	2.97×10^{-4}	2.58

Table S2. Comparison of the lowest electrical percolation thresholds in the literature

Matrix polymer	Filler type	Lowest percolation threshold reported (vot %)	Ref.
Polycarbonate	TEGO	0.14	[1]
Poly(ethylene)	RG-O	0.07	[2]
Poly(ethylene terephthalate)	TEGO	0.47	[3]
Poly(ethylene)	GNP	0.31	[4]
Poly(propylene)	GNP	0.1	[5]
Poly(styrene)	Funct. G-O	0.1	[6]
Poly(vinylchloride)	GNP	0.6	[7]
Cellulose paper	AgNW	0.0053	This work

We used TG analysis to clarify the weight ratio of AgNW in the as-prepared cellulose papers under different dip-coating cycles (0–10 cycles); the results are shown in Fig. S1. The weight loss process can be divided into two processes. The slight weight loss below 300 oC is ascribed to the loss of absorbed water from the product. Then, a significant weight loss occurs between 300 ^{o}C and 500 ^{o}C, attributing to the pyrolysis of cellulose. The content of AgNW in AgNW/cellulose paper was calculated by the TGA data according to the formula (1). In addition, the AgNW area densitythe were evaluated by using eqs 2-4:

$$w_{AgNW}=\frac{b\text{-}a}{1\text{-}a} \quad (1)$$

$$w_{AgNW}=\frac{M_{AgNW}}{M_{AgNW}+M_{paper}} \quad (2)$$

$$d_{AgNW}=\frac{M_{AgNW}/2}{A_{paper}} \quad (3)$$

Where W_{AgNW} is the weight ratio of AgNW, a and b represent the residual weight ratios of the neat cellulose paper and AgNW/cellulose paper after thermal degradation (800°C), respectively. M_{paper} is the weight of neat cellulose paper (26 mg), M_{AgNW} is weight of AgNW, A_{paper} is the surface area of the cellulose paper (22.86×10.16 mm^2). According to calculation, the mass loading of AgNW in the as-prepared cellulose papers is listed in Table 1.

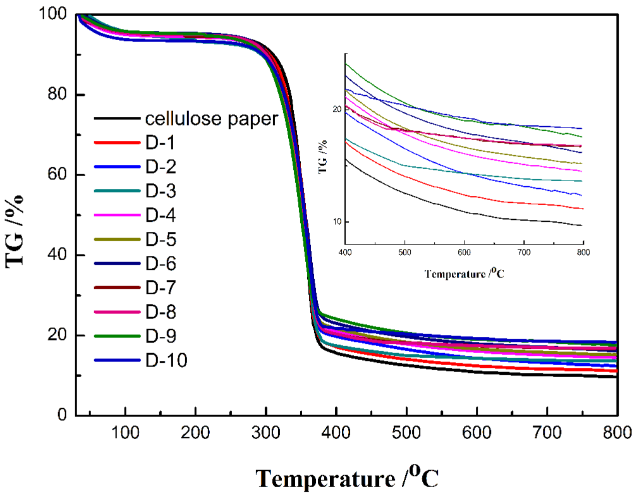

Fig. S1 TG analysis of the as-prepared cellulose papers under different dip-coating cycles (0–10 cycles), measured from 30 to 800 ^{o}C.

The EMI SE values of neat cellulose paper are shown in Figure S2. From Figure S2, almost zero EMI SE indicates that neat cellulose paper has little electromagnetic shielding performance.

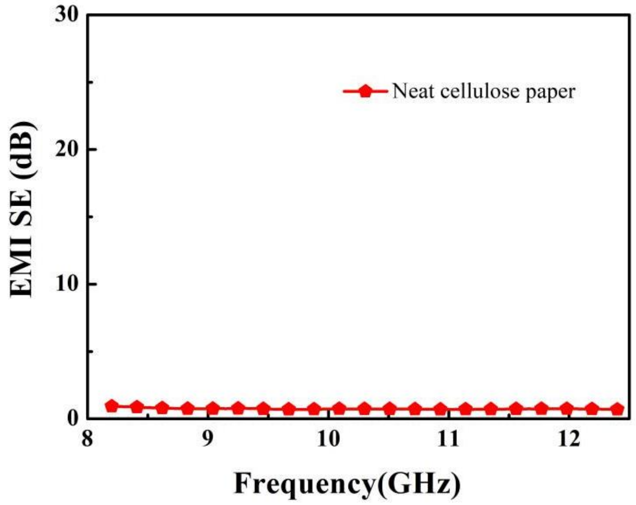

Fig. S2 The EMI SE properties of neat cellulose papers.

The EMI SE values of neat cellulose paper and the sample D-1 before and after hydrophobic modification are shown in Figure S3. From Figure S3, negligible variation on the EMI SE of neat cellulose paper (Figure S3a) and D-1 (Figure S3b) implies the ceramic coating has no effect on the EMI SE of the sample, thus it is concluded that the inorganic ceramic coating does not cause a decrease in conductivity.

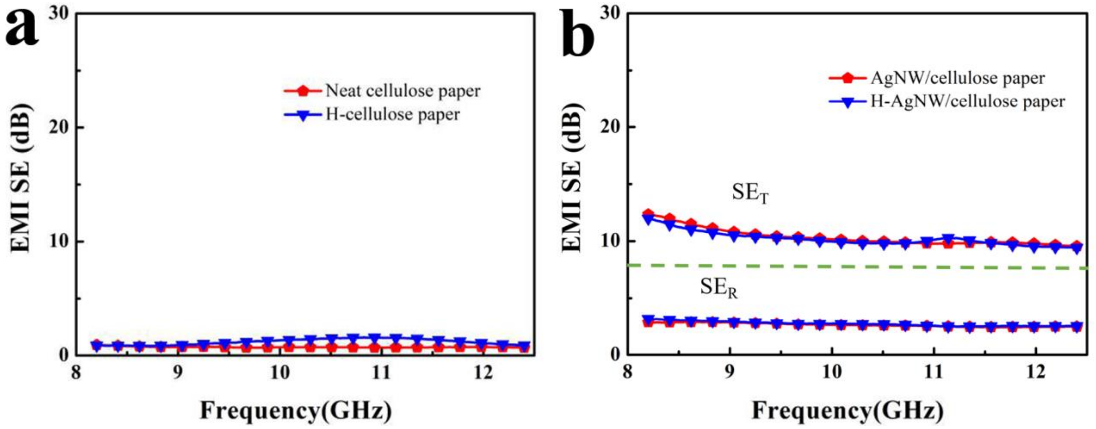

Figure S3. The EMI SE values of neat cellulose paper (a) and the sample D-1 (b) before and after hydrophobic modification.

Similarly, in order to further comprehensively evaluate the EMI shielding durability of the H-AgNW/cellulose papers, EMI SE variation of the papers undergoing other ultrasonic and environmental tests were also investigated, and the corresponding results also displayed in Figure S4. No obvious EMI SE degradation was observed for Dip-9 and the water in the bottle remained clear and transparent after ultrasonic treatment for 60 min (Figure S4a), indicating the mechanical robustness and fastness of the hydrophobic coating. Moreover, negligible variation in static CA over the mechanically perturbed surfaces implies the durability of the coating with environmental effects (Figure S4b).

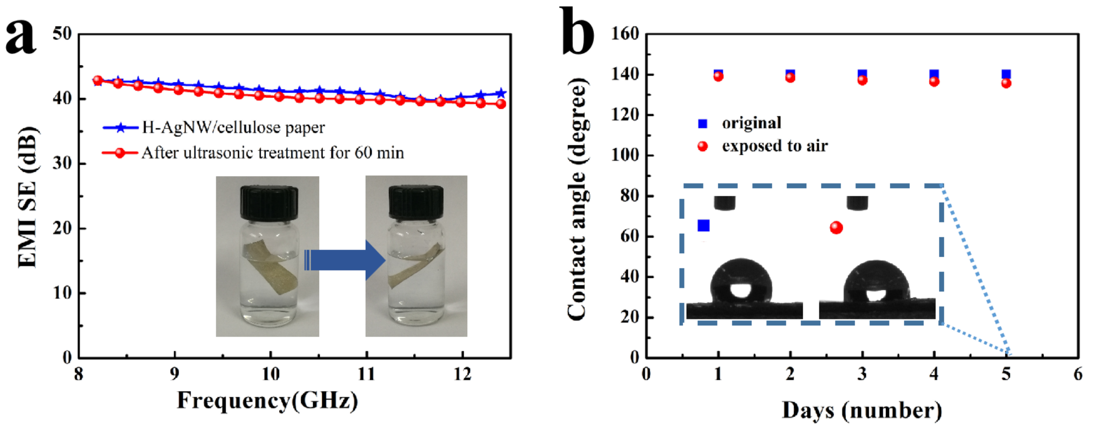

Figure S4. (a) EMI SE in the range of 8.2-12.4 GHz (X band) of the H-AgNW/cellulose after washing three days; (b) Change in CA of water droplet during exposure to air for 5 days (Inset photograph showing static CA of water on mechanically tested surfaces)

The indentation stiffness of the samples and the results is shown in Figure S5. With the dip coating cycles increasing, indentation stiffness of the samples increased slightly, while a large increment was obtained for H-AgNW/cellulose papers. This may be ascribed to the excellent hardness of AgNW and inorganic hydrophobic layer as well as increment in thickness of the the composite papers.

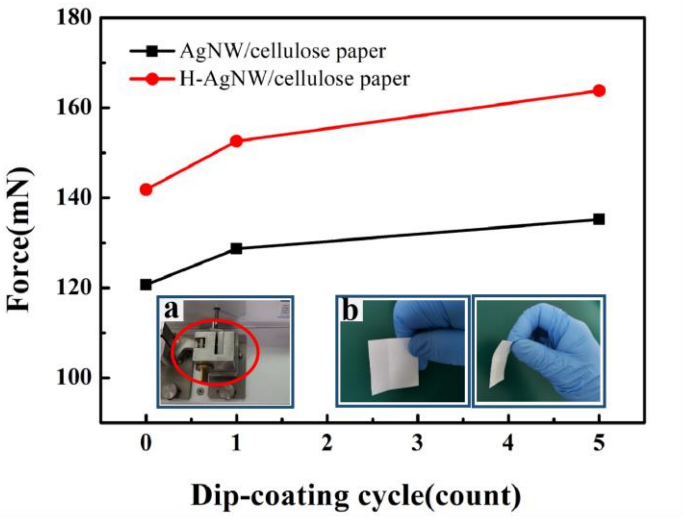

Fig. S5 The stiffness test of the cellulose paper and and the H-AgNW/cellulose paper.

References

1. Yoonessi, M.; Gaier, J.R. Highly Conductive Multifunctional Graphene Polycarbonate Nanocomposites. ACS NANO, 2010, 4, 7211-7220.
2. Pang, H.; Chen, T.; Zhang, G.; Zeng, B.; Li, Z.M. An electrically conducting polymer/graphene composite with a very low percolation threshold. Materials Letters, 2010, 64, 2226-2229.
3. Zhang, H.B.; Zheng, W.G.; Yan, Q.; Yang, Y.; Wang, J.W.; Lu, Z.H.; Ji, J.Y.; Yu, Z.Z. Electrically conductive polyethylene terephthalate/graphene nanocomposites prepared by melt compounding.

Polymer, 2010, 51, 1191-1196.

4. Chen, G.; Weng, W.; Wu, D.; Wu, C. PMMA/graphite nanosheets composite and its conducting properties. European Polymer Journal, 2003, 39, 2329-2335.
5. Kalaitzidou, K.; Fukushima, H.; Drzal, L.T. A new compounding method for exfoliated graphite–polypropylene nanocomposites with enhanced flexural properties and lower percolation threshold. Composites Science and Technology, 2007, 67, 2045-2051.
6. Stankovich, S.; Dikin, D.A.; Dommett, G.H.B.; Kohlhaas, K.M.; Zimney, E.J.; Stach, E.A.; Piner, R.D.; Nguyen, S.T.; Ruoff, R.S. Graphene-based composite materials. NATURE, 2006, 442, 282-286.
7. Vadukumpully, S.; Paul, J.; Mahanta, N.; Valiyaveettil, S. Flexible conductive graphene/poly(vinyl chloride) composite thin films with high mechanical strength and thermal stability. Carbon, 2011, 49, 198-205.
